# Testicular involvement in pediatric acute lymphocytic leukemia: what to do about it?

**DOI:** 10.1590/S1677-5538.IBJU.2022.0318

**Published:** 2022-08-31

**Authors:** Lisieux Eyer de Jesus, Samuel Dekermacher, Glaucia Campos Resende, Renata Rangel Justiniano

**Affiliations:** 1 Hospital Universitário Antônio Pedro Niterói RJ Brasil Serviço de Cirurgia pediátrica, Hospital Universitário Antônio Pedro - Niterói, RJ, Niterói, Brasil; 2 Hospital federal dos Servidores do Estado Rio de Janeiro RJ Brasil Serviço de Cirurgia pediátrica, Hospital federal dos Servidores do Estado - Rio de Janeiro, RJ, Brasil

## INTRODUCTION

Acute lymphocytic leukemia (ALL) represents 30% of childhood cancers (1) and relapsed ALL is the leading cause of death caused by cancer in childhood (2).

Relapse of ALL typically arises from the bone marrow. Extramedullary relapse most commonly involves the central nervous system but may also present as a testicular isolated or concomitant relapse in males (3).

Information about testicular leukemia is difficult to analyze, considering difficulties to compare patients treated with protocols that are not in use contemporaneously to children treated nowadays, the different results of leukemia treatment in developed and low/medium income countries (LMIC) results referring to multiple age groups, the relative rarity of the disease and the limited quality of the literature.

## MATERIALS AND METHODS

We made an analytic descriptive non-systematic literature review about testicular recurrence after ALL in childhood. The key words “leukemia AND (testis OR testicle)” were used to find papers through PUBMED, limited to children (0-18 years old), in Portuguese, Spanish, English, or French. The abstracts were then reviewed. Papers dealing primarily with TR after ALL were reviewed. The selected papers were then read in toto. Any other papers of interest retrievable from the references were also reviewed. Gray literature was not included.

## RESULTS

Testicular involvement in pediatric ALL is rare in patients from developed countries being treated with modern protocols, where more than 90% survive (4), with a proportion of 15-20% relapses after first remission, mostly bone marrow disease (5–6). Lower prevalence of testicular ALL is strongly dependent on modern chemotherapy protocols: testicular recurrences (TR) affected 5.8 to 16.2% of the patients in the ‘70s, versus 0-2% in contemporaneous cohorts from developed countries (0-2%), equally divided between early, intermediate and late occurrences (2, 3, 6, 7). Data from 308 ALL autopsies on leukemia patients from 1958-1982 (88 children) showed involvement of the testes in 15% of the most recent cases (1977-1982) versus 49% of those treated between 1958-1964 (8). Later and intermediate TR usually occurs 2-3 years after remission but has been reported up to 10 years after treatment of myeloid leukemias (9).

TR incidence in LMIC persists similar to that described in the ‘70s, mainly due to late diagnosis and irregular or inadequate chemotherapy, despite the adoption of intermediate/high-dose methotrexate (MTX) protocols. In India, almost a quarter of ALL children recur (half of those early), and a quarter of the relapses present TR (equally divided between isolated and concurrent) (10).

Timing of TR (early/< 18 months, intermediary/18-35 months or late/≥ 36 months after remission), kind of relapse (isolated versus concurrent TR), and the number of relapses presented by the patient are directly related to the prognosis. Early, concurrent, and successive relapses are related to a worse prognosis. TR is partially responsible for the worse prognosis of ALL in males as compared to females (11).

Studies from autopsies of ALL patients, obviously biased towards severe and/or irresponsive cases, report frequent testicular involvement (25-48.5% of the patients) associated with other disease foci (12, 13), suggesting that the testis may be frequently involved in disseminated uncontrolled disease and that testicular leukemia that is associated to severe disseminated early or recurrent leukemia may be different from TR, especially if isolated.

Three different clinical patterns of TR may be seen:

1. Isolated TR;
2. Concurrent relapse associating TR to disease attaining other loci;
3. Advanced/out of control severe leukemia.

The efficacy of chemotherapy on the testicles is limited due to insufficient penetration of some drugs in the blood-testicular barrier (“pharmacological sanctuary”). Although the physiological basis for a blood-testis barrier is unclear, it has been demonstrated that some systemic medications do not distribute equally to the testes. The concentrations of methotrexate (MTX) are 2-4 times lower in the testicular interstitial space (the typical site of tumoral invasion) and 18-50 times lower in the tubules (typically associated with late tumoral invasion) than in the patient's blood MTX (6, 14). This can be resolved with higher doses of MTX (14), prolonged chemotherapy, and usage of vincristine and/or cyclophosphamide (drugs that surpass the blood-testicular barrier) associated with steroids. Departing from this information, the usage of intermediate/high intravenous doses of MTX from the 1980s significantly lowered the incidence of TR. Some authors also suggest that the lower temperatures typical for normally located testis may also be related to the lower pharmacological activity of some drugs (15).

Prepubertal and peripubertal testicles may respond differently to neoplastic invasion, as compared to mature gonads (16). Cases presenting next to puberty may be more prone to TR than pre- or post-pubertal patients (6, 11). Immunological tolerance to neoplastic cells may also differ in the testicles as compared to other organs, locally “protecting” and “harboring” leukemia cells (4, 11). Two possibilities involving relapses have been suggested: (1) development from original clones that survived chemotherapy and are probably chemoresistant; (2) isolated or non-isolated TR developing from cells from a common ancestral clone previously protected from chemotherapy as a testicular nidus (2). This has obvious implications for treatment, with potentially worse results with rescue chemotherapy for chemotherapy-resistant disease, and calls for more studies (4, 16).

The involvement of the testicle in ALL cases is more frequent in high-risk patients (< 1-year-old, ≥ 10 years old, patients presenting higher peripheral blasts counts at the moment of the diagnosis, T-cell leukemia cases showing significant hepatomegaly/splenomegaly or > 2 cm lymph nodes, unfavorable genetic patterns (BCR-ABL1 and KMT2A), and multiple recurrences) (1, 4, 10). Those need aggressive treatment, frequently including bone marrow transplantation (4).

Testicular ALL may (1) be simultaneous to the diagnosis of the primary disease (1.1 to 2.4% of the patients), and (2) present after treatment and remission of the primary disease (TR).

**Figure 1:**
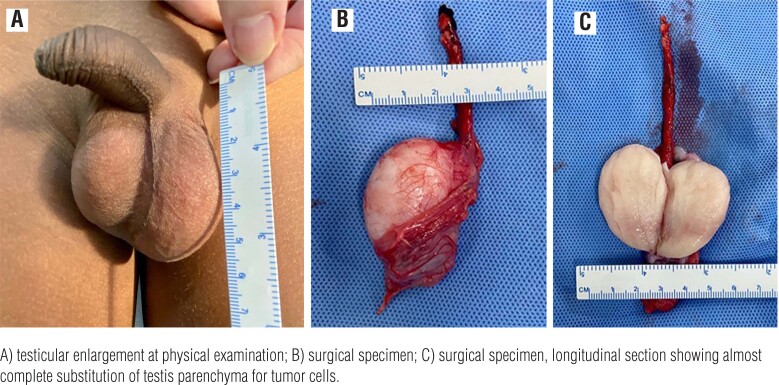
Early left testicular recurrence in a 4-year-old boy.

Clinically, the involved testis is usually augmented (Figure-1), irregular, firmer than a normal testicle, and usually painless. Bilateral enlargement is typically asymmetric. Discoloration may be rarely seen. The growth rate is quite rapid: some authors report that many of the involved testicles doubled in volume in 15 days (3).

In ultrasound, the testis is diffusely hypoechogenic or presents hypoechogenic nodules that are hypervascular on Doppler (17). The role of 18F FDG PET/CT to detect extramedullary leukemia is still being studied. Most data are available from relapsed patients, with sensitivity, specificity, and accuracy of, respectively, 93.3%, 71.4%, and 79.7%. The exam was able to detect all 4 cases of TR in Zhou's paper, 3/4 as diffuse uptaking/involvement. No false positives (usually related to inflammatory or infectious complications) were reported (9).

Fine needle and core needle biopsies are both accurate for diagnosis. Needle biopsy specimens provide results that are similar to open wedge biopsies (18–21). Microscopically the involved testicle shows focal or diffuse infiltration of leukemic blasts with effacement of the normal architecture (22), frequently arranged in a concentric fashion around tubules or vessels in the interstitial tissue (7). Tubular involvement is a late event (4). The specimen should be handled with care to avoid crushing artifacts and should be promptly fixed. There is some controversy about what concerns the fixative (formaldehyde, Bouin's, or Zenker's) (23).

“Isolated” TR should be considered a systemic disease: exclusive local treatment is associated with early systemic recurrence, including 10-20% with central nervous system invasion. Concerning treatment:

1. Clinically apparent testicular involvement simultaneous to primary diagnosis/treatment is rare (less than 2% of the patients) and is treated as “common” cases. This clinical presentation is related to severe disease at presentation (hepatosplenomegaly, lymphonodomegaly, high number of peripheral blasts), but the results of treatment are similar to those in other cases in modern cohorts: testicular involvement in the initial clinical presentation is not an individual predictive factor (24, 25). Testicular irradiation is used only if the testicular abnormalities persist after chemotherapy. This is controversial and some authors advocate for local radiotherapy depending on the moment of the diagnosis, the bulk of testicular disease, and initial response to chemotherapy or for post-chemotherapy “control” biopsies by protocol (1). Subclinical testicular involvement normally responds to routine modern chemotherapy protocols. So, there is no need to actively search for testicular foci in ALL patients presenting clinically normal testicles and a normal ultrasound examination (4, 26).
  - Systematic testicular biopsy just before discontinuing maintenance therapy in patients in remission was routine till the 1980s. Positive biopsies led to bilateral testicular irradiation and extension of chemotherapy in positive cases) (23). This was abandoned, considering the better results of modern chemotherapy protocols, the relative rarity of TR, the possibility of false negatives, late diagnoses of TR despite negative biopsies, and results similar to those found in patients diagnosed departing from clinical symptoms/signs of TR (4, 27–30).
  - Prophylactic testicular irradiation has been abandoned, in order to minimize gonadal dysfunction and infertility (14, 31), as no improvement in survival has been demonstrated in patients that received adequate systemic chemotherapy (32).
2. Post-remission TR:
  - Local treatment: bilateral gonadal irradiation (24 Gy/2500 rads distributed in 2 weeks, 12 sessions, applied to the testis and spermatic cords (31, 33) or orchiectomy (generally reserved for unilateral cases and patients who refuse radiotherapy) + contralateral prophylactic irradiation (15 Gy), trying to preserve Leydig function and the potential for spontaneous puberty. However, lower testosterone levels in response to HCG stimulation, as compared to normal children/adolescents, have been proved after testicular irradiation (34), as well as delayed or arrested spontaneous puberty, and high serum FSH (31). Contralateral irradiation is needed even if the contralateral testicle seems normal clinically, as microscopic involvement is common: unilateral orchiectomy is not sufficient, but may be done with lower doses (15 Gy) (3). The penis is taped away from the irradiation field in order to protect the urethra. The responsive testes reduce to the normal size during the first month after treatment (3). Associated intensive re-induction chemotherapy with high doses of MTX is mandatory.
  - Late TR after bone marrow transplantation: orchiectomy is mandatory (4, 35), but other treatments are controversial, considering previous exposures to chemotherapy and bad prognosis (36).
  - There is no published protocol specifically considering the technique for orchiectomy in ALL TR. The concept of avoiding lymphatic spreading of the tumor via scrotal incisions seems to apply, and trans-inguinal radical orchiectomy seems appropriate, as done in primary testicular tumors.

Immunotherapy using CD19-specific chimeric antigen receptor T (CAR-T) cells has shown good results and complete remission in 6/7 patients with isolated TR (5-23 months follow-up), without radiotherapy or orchiectomy. One patient re-recurred (bone marrow), but all showed a complete testicular response (37).

The prognosis varies with the timing of TR: 5 years of survival varies from 13.6% in early recurrences (< 18 months) to 52.2% (18-36 months) and 60% (late recurrences, ≥ 36m after treatment), with modern treatment protocols (4).

## DISCUSSION

The quality of the information about TR after ALL is a serious problem. Most papers about the TR are from the ‘80s and the ‘90s and report on retrospective descriptive cohorts. Many mix children and adults, leukemias and lymphomas, and different types of leukemia together. Also, papers from LMIC are scarce. Most information available comes from developed countries, which usually present more favorable results, and provide easy access to state-of-the-art treatments. This is directly related to the high indexes of TR in LMICs when compared to developed countries, but the effect of worse treatments is difficult to separate from the effect of late diagnoses and the predominance of more advanced cases in LMICs. Also, the protocols may have to be adjusted to the circumstances. Considering the relatively high incidences of TR, would it be useful to adopt post-remission testicular biopsies in LMICs, despite this having been eliminated from developed countries’ protocols?

Would it be useful to frequently follow up the testis with imaging after remission? As some suggest, testicular “harbored” blasts may be the origin of systemic leukemia relapse. Would early detection of subclinical testicular nodules facilitate treatment?

Some clinical situations are exceedingly rare (e.g. TR after bone marrow transplantation), making it difficult to evaluate and compare the treatment strategies and establish reasonable treatment protocols.

Basic research is needed to understand the testicular-blood barrier in detail. This may deepen our understanding of the immunological response of the testis to leukemic cells and, potentially, suggest new methods of treatment and avoidance of future TR.

**Figure 2:**
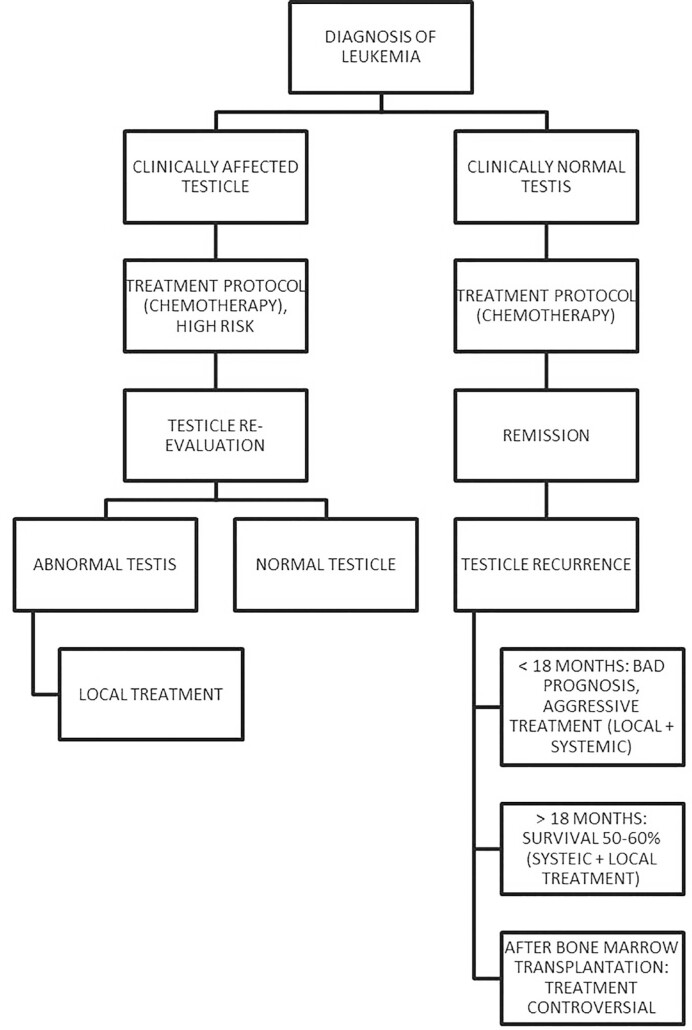
Treatment algorithm suggested by the authors to treat ALL cases, according to the clinical situation and response to treatments, considering primary testicular affection and testicular recurrence. Local treatment in this context means either ipsilateral orchiectomy or radiotherapy with contralateral biopsy.

A recent paper describing excellent results of immunotherapy may be a first suggestion that this may also be used to avoid future TR in the future (37). TR, despite being treatable, leads to prolonged chemotherapy, high risks of infertility, and frequent need for external androgens in young adults. Survival after early TR remains dire. New protocols to avoid and treat TR are needed. We suggest herein a treatment algorithm, based on the current state of the art (Figure-2).

In conclusion, testicular affection by ALL is not rare in LMICs, despite being very uncommon in developed nations. Data about this condition are mainly based on results from developed countries in the 20th century, which may not apply to the contemporaneous circumstances found in LMIC. TR worsens prognosis, and is related to late diagnosis of ALL, suggesting that better pediatric health care may help to get earlier diagnoses and to diminish the incidence of testicular ALL. Also, more research is needed on this subject, especially concerning physiopathology and the role of the testes as “leukemia sanctuaries” and about the promising new proposals for the immunological treatment of ALL testicular relapses.

## ABBREVIATIONS

ALL =: acute lymphocytic leukemia
LMIC =: low and middle-income country
TR =: testicular recurrence
